# Mechanical Explanation for the Hemodynamic Efficacy of Right Atrial Appendage Autografts Used in Neo–Semilunar Valve Leaflet Reconstruction

**DOI:** 10.1016/j.atssr.2025.04.021

**Published:** 2025-05-16

**Authors:** Andrew Behrmann, Kate Appleman, Katarina Lettner, Balaji Cherupala, Jessica Cayton, Shamik Bhattacharya, Pirooz Eghtesady

**Affiliations:** 1 School of Medicine, University of Missouri, Columbia, Missouri; 2 Department of Engineering, Southeast Missouri State University, Cape Girardeau, Missouri; 3 Department of Veterinary Pathobiology, University of Missouri, Columbia, Missouri; 4 Department of Cardiothoracic Surgery, Washington University School of Medicine, St Louis, Missouri

## Abstract

**Background:**

Pediatric pulmonic and aortic valve insufficiency has recently been treated with novel procedures that use autologous right atrial appendage tissue. Whether atrial tissue’s tensile strength correlates with pressures it can withstand to function as a neo–semilunar valve has not been studied. We therefore studied these properties using an ex vivo porcine heart model.

**Methods:**

Aortic cusps, pulmonic cusps, and right and left atrial appendage tissues were excised from 10 porcine hearts. Native leaflets and atrial appendage samples underwent circumferential and radial stretching by a uniaxial tensile machine. Right atrial appendage tissue from 14 additional porcine hearts was used to reconstruct neo-pulmonic and neo-aortic valves that were hydrostatically pressurized until failure.

**Results:**

The modulus of elasticity was significantly greater in aortic (3.11 ± 0.53 MPa circumferential; 1.31 ± 0.29 MPa radial) and pulmonic (2.99 ± 0.46 MPa circumferential; 1.12 ± 0.24 MPa radial) cusps than in right (0.36 ± 0.06 MPa circumferential; 0.30 ± 0.04 MPa radial) and left (0.41 ± 0.07 MPa circumferential; 0.36 ± 0.06 MPa radial) atrial appendage tissues. The average hydrostatic pressure at valve failure was similar between neo-pulmonic (104.9 ± 4.9 mm Hg) and neo-aortic (102.7 ± 2.4 mm Hg) valves.

**Conclusions:**

Atrial appendage tissue’s greater distensibility may allow greater leaflet coaptation and accommodate expansion over time, enhancing growth potential. However, the greater pliability may predispose to valve prolapse if too much tissue is used or in the setting of elevated diastolic pressures. Longitudinal studies are warranted to further explore the potential of atrial tissue for valve reconstructions.

**In Short**

▪ Atrial appendage tissue is significantly more distensible than native aortic and pulmonic cusps.
▪ Despite their distensibility, atrial appendage valves can withstand elevated diastolic pressures in the in vitro setting.

Congenital heart defects involving aortic and pulmonic valves present significant challenges in pediatric cardiothoracic surgery because of the high frequency of reoperations. In tetralogy of Fallot (ToF), various techniques, including autologous pericardial valve reconstruction, homograft, and valve-sparing methods, have been used, but they still carry a risk of right ventricular failure or reoperation.^1^ A critical consideration for pediatric valve replacements is that they must accommodate somatic growth. There has been increasing interest in using right atrial appendage (RAA) tissue to create neo–pulmonary valves during right ventricular outflow tract reconstruction in ToF repair, showing favorable hemodynamic outcomes.^2^ Other procedures that have used atrial tissues for reconstruction of atrial septal defect or right ventricular outflow tract enlargement displayed viable subendocardial and subepicardial myocytes and a lack of desmin immunoreactivity.^3^ Thus, autologous atrial tissue used in reconstruction remains viable and may offer growth potential without rejection risk. These properties and promising results in ToF repair have led to its use in other valve reconstructions as well.^4^, ^5^, ^6^ Studying the mechanical properties of materials used for valve reconstruction is key to understanding optimal implantation techniques and potential durability and function. This study provides the first direct comparison of RAA and left atrial appendage (LAA) tissue to native semilunar valve cusps to evaluate the potential of atrial tissue as neo–semilunar valve leaflets.

## Material and Methods

**Figure 1:**
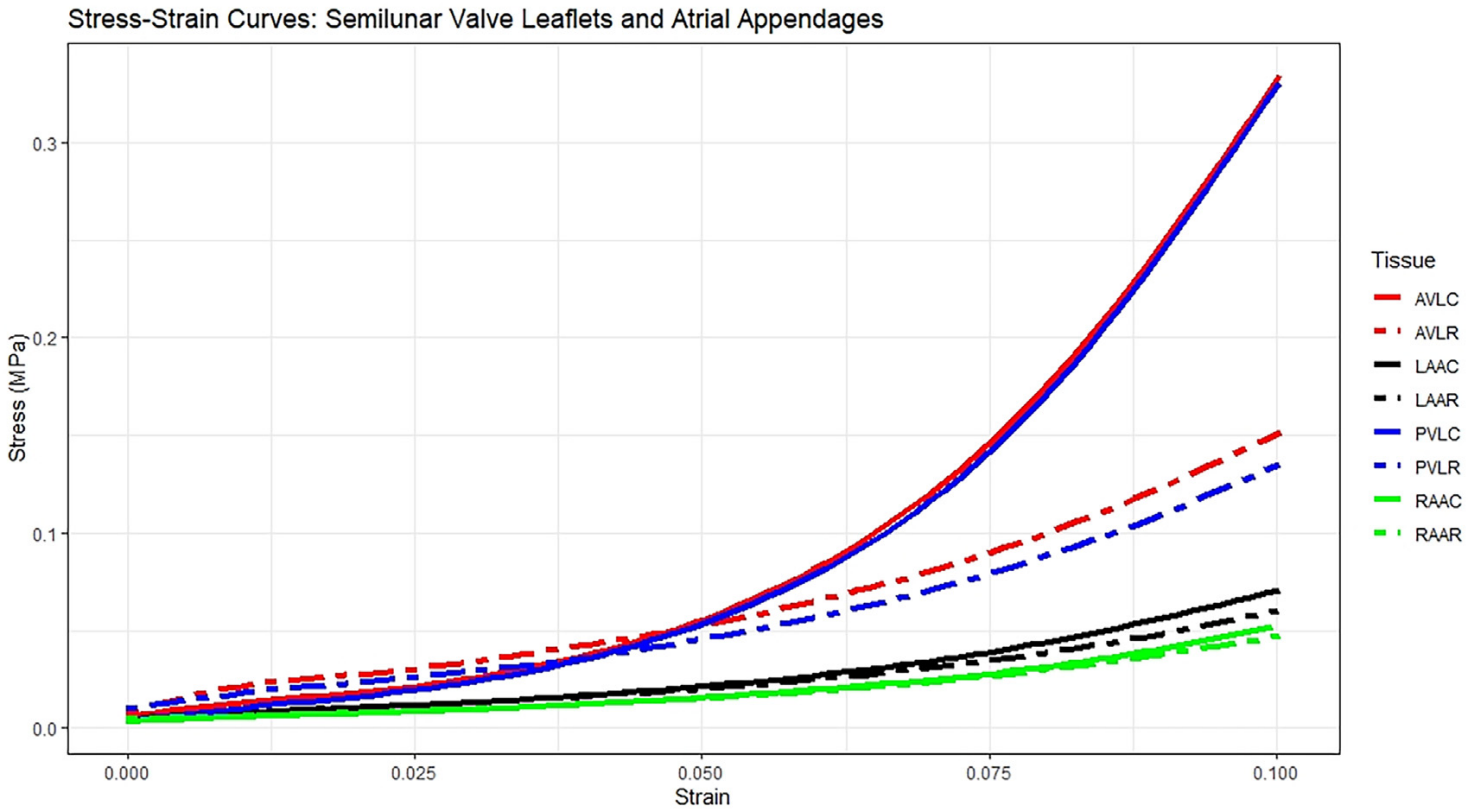
Averaged stress-strain curves generated from porcine semilunar valves and atrial appendage tissues. (AVLC, aortic valve leaflet circumferential; AVLR, aortic valve leaflet radial; LAAC, left atrial appendage circumferential; LAAR, left atrial appendage radial; PVLC, pulmonary valve leaflet circumferential; PVLR, pulmonary valve leaflet radial; RAAC, right atrial appendage circumferential; RAAR, right atrial appendage radial.)

Ten porcine hearts were cryopreserved at −80 °C and thawed in 37 °C saline on the experiment day. Atrial appendage tissues and native valves were harvested, with pectinate muscles removed. Two 1 × 1-cm samples were cut from the belly region of pulmonic, aortic, and atrial tissues, oriented circumferentially or radially for uniaxial tensile testing. Samples were mounted on the UStretch (CellScale) tensile machine in 37 °C saline, preconditioned with 5 cycles of 10% strain at 0.25 mm/s, and stretched to 10% strain at 0.1 mm/s to generate stress-strain curves (Figure 1). Fourteen additional hearts underwent mock pulmonic and aortic valve reconstructions using RAA tissues for bicuspid neovalves. Neovalves were pressurized retrogradely with 37 °C saline until failure, defined by a drop in pressure.

## Results

**Figure 2:**
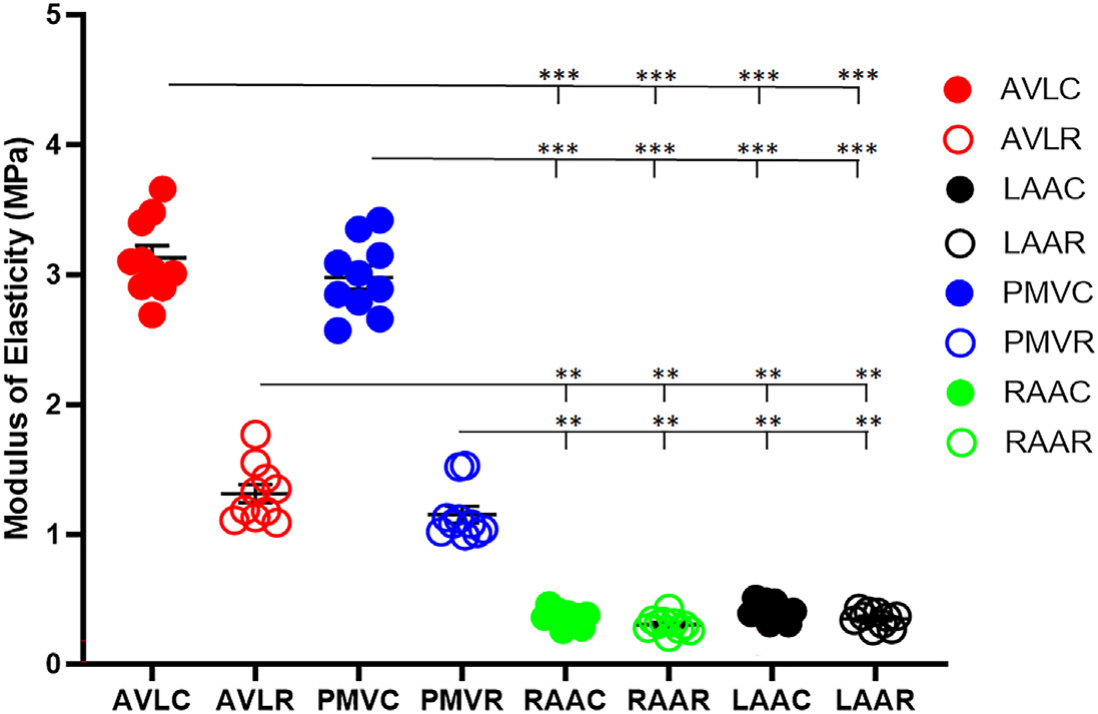
Average modulus of elasticity values ± SD of tissues stretched radially or circumferentially. ∗∗*P* < .001. ∗∗∗*P* < .0001. (AVLC, aortic valve leaflet circumferential; AVLR, aortic valve leaflet radial; LAAC, left atrial appendage circumferential; LAAR, left atrial appendage radial; PMVC, pulmonary valve circumferential; PMVR, pulmonary valve radial; RAAC, right atrial appendage circumferential; RAAR, right atrial appendage radial.)

**Figure 3:**
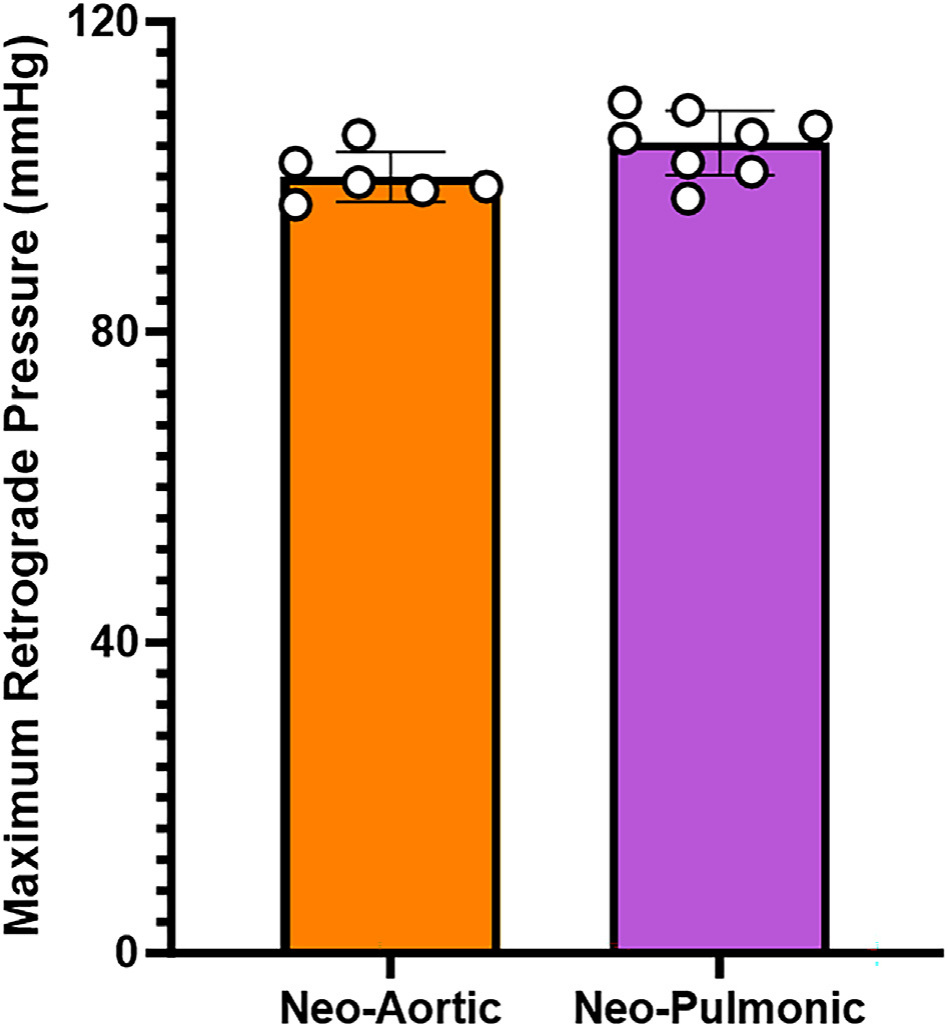
Average hydrostatic pressure at which neo-aortic and neo-pulmonic valves underwent failure.

The modulus of elasticity was significantly greater in aortic cusps (3.11 ± 0.53 MPa circumferential; 1.31 ± 0.29 MPa radial) and pulmonary cusps (2.99 ± 0.46 MPa circumferential; 1.12 ± 0.24 MPa radial) than in RAA (0.36 ± 0.06 MPa circumferential; 0.30 ± 0.04 MPa radial) and LAA tissues (0.41 ± 0.07 MPa circumferential; 0.36 ± 0.06 MPa radial) in both circumferential and radial dimensions (Figure 2). After discovering the differences in tensile strength, we determined at what pressures the RAA valves would fail in an in vitro model. The average pressure at valve failure of the neo-pulmonary (n = 8) RAA valves was 104.9 ± 4.9 mm Hg; the average pressure at failure of the neo-aortic (n = 6) RAA valves was 102.7 ± 2.4 mm Hg (Figure 3).

## Comment

This study highlights significant differences in tensile strength between semilunar valve cusps and atrial appendage tissues. Consistent with previous findings, pulmonic and aortic cusps showed similar anisotropic mechanical properties. In contrast, atrial tissue exhibited pliable, isotropic behavior, which could simplify surgical implantation by eliminating the need for specific orientation to mimic native valve mechanics. These properties may also enhance durability, improve resistance to mechanical fatigue, and better accommodate variable hemodynamic stresses. In addition, the isotropic nature of atrial tissue may allow it to function effectively across various valve designs (eg, semilunar vs atrioventricular) without requiring significant modifications. The similar mechanical properties of RAA and LAA tissues suggest that LAA may be a viable alternative for valve reconstruction in patients with unfavorable RAA anatomy.

The ideal valve reconstruction material remains unknown. Autologous pericardium is prone to calcification and lacks growth potential. Glutaraldehyde treatment reduces calcification but renders the tissue nonviable. Bioprosthetic valves shift from anisotropic to isotropic mechanics before failing. It is unclear whether this applies to atrial tissues as they naturally exhibit isotropic mechanics and greater pliability. However, atrial tissues may differ because of distinct cellular composition/ultrastructure compared with pericardium,^7^ demonstrated viability when used in reconstruction,^3^ and their distensibility, which may allow growth while maintaining proper leaflet function.

Increased pliability of atrial tissue may cause valve prolapse or regurgitation if excessive tissue is implanted or under excessively high diastolic pressures, as shown in our hydrostatic pressurization experiments. Native valves withstand higher pressures (120 mm Hg for pulmonic and 190 mm Hg for aortic) than atrial tissue valves in this study, emphasizing the need for normal blood pressures in patients.^8^^,^^9^ The long-term viability and remodeling of atrial appendage tissue for valve reconstruction remain uncertain, warranting further investigation into how valve geometry and commissure length affect durability and function.

Neovalves were tested in porcine outflow tracts and may not reflect clinical conditions. Pressure studies were conducted hydrostatically; dynamic testing might yield different results. Future studies could use biaxial tensile testing for more comprehensive insights into RAA tissue behavior.
